# Practicability of Hygienic Wrapping of Touchscreen Operated Mobile Devices in a Clinical Setting

**DOI:** 10.1371/journal.pone.0106445

**Published:** 2014-09-02

**Authors:** Matthias Hammon, Bernd Kunz, Veronika Dinzl, Ferdinand J. Kammerer, Siegfried A. Schwab, Christian Bogdan, Michael Uder, Philipp M. Schlechtweg

**Affiliations:** 1 Department of Radiology, University Hospital Erlangen, Erlangen, Bavaria, Germany; 2 Mikrobiologisches Institut - klinische Mikrobiologie, Immunologie und Hygiene, Friedrich-Alexander-Universität Erlangen-Nürnberg, Erlangen, Bavaria, Germany; The University of Tokyo, Japan

## Abstract

**Background:**

To prove effectiveness of wrapping tablet computers in order to reduce microbiological contamination and to evaluate whether a plastic bag-covered tablet leads to impaired user satisfaction or touchscreen functionality.

**Materials and Methods:**

Within a period of 11 days 115 patients were provided with a tablet computer while waiting for their magnetic resonance imaging examination. Every day the contamination of the surface of the tablet was determined before the first and after the final use. Before the device was handed over to a patient, it was enclosed in a customized single-use plastic bag, which was analyzed for bacterial contamination after each use. A questionnaire was applied to determine whether the plastic bag impairs the user satisfaction and the functionality of the touchscreen.

**Results:**

Following the use by patients the outside of the plastic bags was found to be contaminated with various bacteria (657.5 ± 368.5 colony forming units/day); some of them were potentially pathogenic. In contrast, the plastic bag covered surface of the tablet was significantly less contaminated (1.7 ± 1.9 colony forming units/day). Likewise, unused plastic bags did not show any contamination. 11% of the patients reported problems with the functionality of the touchscreen. These patients admitted that they had never used a tablet or a smartphone before.

**Conclusions:**

Tablets get severely contaminated during usage in a clinical setting. Wrapping with a customized single-use plastic bag significantly reduces microbiological contamination of the device, protects patients from the acquisition of potentially pathogenic bacteria and hardly impairs the user satisfaction and the functionality of the touchscreen.

## Introduction

It is well known that colonizations and infections with microbial pathogens have an important impact on health care systems. They can cause clinical problems and raise costs. Bacteria increasingly develop resistance against various antibiotics. Therefore, both the employees and the patients in a hospital have to be protected from acquisition of potentially pathogenic bacteria as much as possible.

Since the introduction of the iPad by Apple Inc. (Cupertino, California, USA) in 2010, modern tablet computers (tablets) have become increasingly popular. One reason for the broad acceptance of the tablets is their compact design, portability and user-friendliness. These properties make tablets attractive for medical or research applications [1]–[7]. Their intuitive interface with direct on-screen interaction, the growing supply with a variety of applications and their connection to the internet suggests usage for different clinical purposes. These portable devices can assist physicians in the recording of patients' data, in decision making by providing clinical algorithms and rapid access to data bases, in obtaining informed consent from patients, or in getting a second opinion from experts. In addition, patients can also benefit from tablets, for example during waiting periods, when these portable computers provide access to valuable information on diseases and the respective diagnostic and therapeutic procedures or allow for short-term entertainment.

In previous publications on the use of mobile examination instruments (e.g. stethoscopes) and communication devices (e.g. cellular phones and pagers) in hospitals bacterial contamination and aspects of hygiene have been a matter of concern [8]–[10]. In order to ensure hygienic conditions and to prevent microbial contamination and spreading, it is crucial either to clean or protect these devices with exchangeable plastic bags or foils. The bacterial load on the surface of tablet computers could be decreased by disinfection [11]. However, disinfection of these touch-sensitive devices with a leaky, non-protected case does not seem to be straightforward. Manufacturers like Apple Inc. recommend to use only a soft, lint-free cloth to clean the surface of the tablet. Abrasive cloths, towels, paper towels and similar items may cause scratches to the item [12], [13]. Agents such as window- or household cleaners, aerosol sprays, solvents, alcohol, ammonia, or abrasive cleaners should not be used [12], [13]. The product warranty of the company expires immediately when only a small amount of liquid reaches the interiority of an Apple product [12], [13]. These hygienic limitations complicate the use of tablets in a hospital environment.

This study was set up to determine whether wrapping in a dedicated, single-use plastic bag reduces microbial contamination of a tablet computer during usage in a clinical setting. Therefore we investigated the extent of the microbial contamination of the surface of the device and of the outside of the plastic bags before and after usage. Additionally, we evaluated whether the covering plastic bag impairs the user satisfaction and the functionality of the touchscreen.

### Research Highlights

*Tablet computers get severely contaminated during usage in a clinical setting.*
*Plastic bags significantly reduce microbial contamination of touchscreen operated devices.*
*Wrapping protects the user from the acquisition of potentially pathogenic bacteria.*
*Wrapping hardly impairs the functionality and user satisfaction of these devices.*
*Patients who reported operability problems had never used a touchscreen operated device before.*

## Materials and Methods

### Ethics Statement

This study was conducted in accordance with the guidelines of the Declaration of Helsinki and approved by the Ethics Committee of the University Hospital of Erlangen. Written informed consent was obtained from all patients.

### Tablet Computer

A commercially available iPad (version 3) was used. Connections to the internet as well as 16 free of charge applications (games, lifestyle, sports and news) were provided.

### Replicate Organism Detection and Counting (Rodac) Plates

Contact plates, i.e. Rodac plates with a contact area of 24 cm^2^, were used for microbiological monitoring of the plastic-bags and the detection of microorganisms. The Rodac plates were prepared in the Microbiology Institute using contact-dishes (Greiner Bio-One, Frickenhausen, Germany) filled with Tryptone Soya Agar (CASO) (Oxoid Limited, Basingstoke United Kingdom). This culture medium supports growth of a broad spectrum of microorganisms, especially many bacteria, yeasts and moulds. Freshly prepared, unused Rodac plates were delivered daily to the Department of Radiology and were stored in a dedicated refrigerator at 2–8 °C for less than 12 h.

### Plastic Bags

Custom made plastic bags of 26 x 21 cm (TauMedIT GmbH, Laufach, Germany) were used for wrapping the tablet. Prior to the inclusion of the first patient 4 randomly selected unused plastic bags were sampled with Rodac plates to determine their contamination.

### Concept of Study

**Figure 1:**
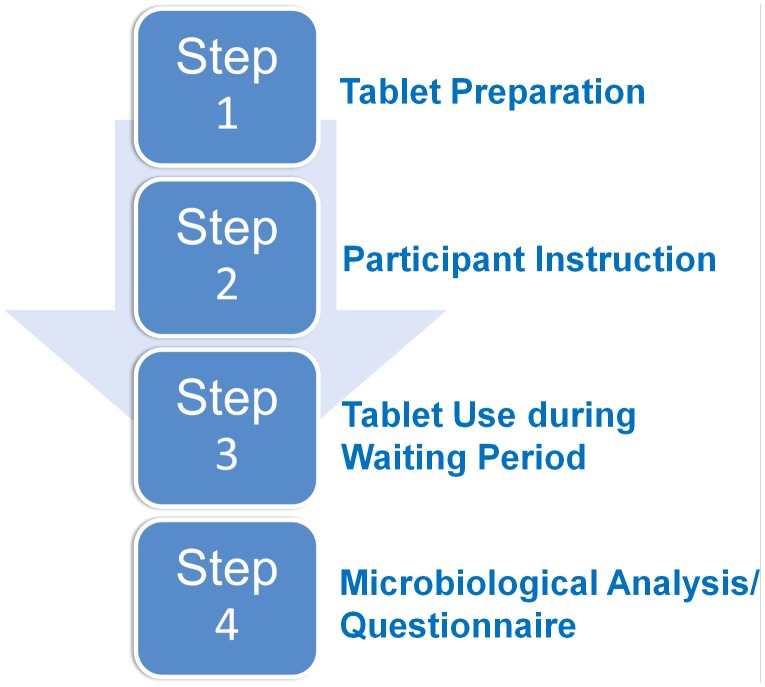
Successive steps of the procedure.

The procedure can be divided into 4 successive steps (Figure 1).

During the whole procedure (preparation of the device, handing out and receiving the device, sample collection), the study assistant handled the device with sterile gloves (Sempermed Supreme, Semperit Technische Produkte GmbH, Vienna, Austria). During the preparation of the tablet and the sample collection the device was always put on a sterile drape cloth of 75 x 90 cm (Foliodrape, Paul Hartmann AG, Heidenheim, Germany).

#### Step 1: Tablet Computer Preparation

Before handing over the tablet to the first patient of the day, the surface of the device was cleaned with Incidin Plus 0.5% (containing glucoprotamine 2-phenoxyethanol and 2-(2-butylethoxy)ethanol; Ecolab Inc., Vienna, Austria) soaked fleece wipes (Pursept Wipes, Merz Hygiene GmbH, Frankfurt, Germany) following the instructions given by the iPad app “deBac” [11].

**Figure 2:**
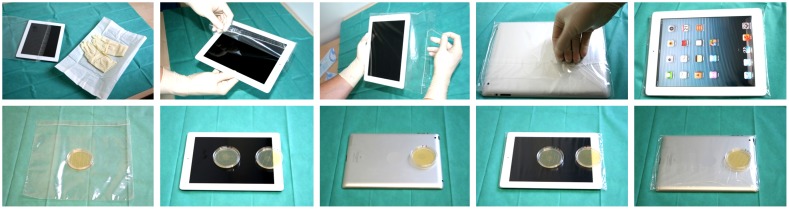
Illustration of the wrapping of the tablet and the sample collection. Upper row illustrates wrapping of the tablet computer using a sterile drape cloth and sterile gloves (Step 1). Lower row illustrates sample collection from an unused plastic bag, from the surface of the tablet and from the outside of a used plastic bag using Rodac plates (Step 1 and 4). The Rodac plates are positioned on the three defined regions of interest (ROIs; ROI A  =  central touchscreen, ROI B  =  home button and its adjacent area, ROI C  =  upper central backside).

After the disinfection agent had evaporated, the microbial contamination of the tablet was assessed each day before initial usage. Probes were taken with Rodac plates from three regions of interest (ROIs), named A, B and C on the surface of the tablet. These 3 ROIs were selected because they are expected to exhibit a high bacterial load. The chosen ROIs were the center of the touchscreen (A), the commonly frequently used home button and its adjacent area (B) and the upper central backside (C) (Figure 2). Afterwards the tablet was placed into an unused plastic bag which was sealed like an envelope. The study assistant wrapping the tablet computer used a sterile drape cloth and sterile gloves to avoid any secondary contamination of the tablet and the plastic bag.

#### Step 2: Participant Selection and Instruction

Within a period of 11 days a study assistant recruited consecutive patients who were referred to the Department of Radiology for magnetic resonance imaging (MRI). Every patient who was neither sedated nor mentally/physically affected was asked to participate in this study. 115 consecutive patients fulfilling the requirements were included. Patients were asked whether they needed an instruction how to handle the iPad. If required the study assistant briefly explained how to use the device and handed over a disposable custom made instruction manual.

#### Step 3: Tablet Computer Use during Waiting Period

The tablet computer enclosed in the single-use plastic bag was handed over to the patient for the entire period of time waiting for the MRI examination. Patients were seated in the waiting area. The tablet could be used at their convenience. When the patients were called to prepare for the MRI examination they returned the tablet to the study assistant.

#### Step 4: Sample Collection/Questionnaire

After the participant returned the device it was handled with sterile gloves and put on a sterile drape cloth. Samples were taken with Rodac plates from the three defined ROIs (ROI A  =  central touchscreen, ROI B  =  home button and its adjacent area, ROI C  =  upper central backside, Figure 2) of the plastic bag covering the tablet. Then the tablet was unpacked and wrapped with a new plastic bag in the same way as described above. There was no additional disinfection of the tablet during the day. For each patient a new, unused plastic bag was taken. Every day, after the final usage of the tablet, samples were not only taken from the plastic bag as described above, but also directly from the surface of the unpacked tablet focusing on the same three ROIs. This procedure allowed to detect or to exclude contamination of the tablet computer itself.

Every patient was asked to complete a questionnaire that requested information about demographics (age, gender) as well as previous experience with a personal computer and touchscreen operated devices (never, occasionally, often, and very often). Additionally, every user was asked whether the plastic bag had impaired the user satisfaction or the functionality of the touchscreen (yes, no) and was invited to value the availability of the tablet during the waiting period (very good, good, fair, and poor).

### Processing of Rodac Plates

After the Rodac plates had been used for contact sampling they were marked with the study number and ROI and were stored in a dedicated refrigerator at 2 – 8 °C for less than 12 h before transfer to the Microbiology Institute. During transportation the plates were packed in an isolated bag. The transport took about 5 minutes (850 meters). In the Microbiology Institute the plates were incubated at 36 °C ± 1 °C for 48 hours under aerobic conditions. After incubation the plates were read by a specialist in microbiology and hygiene. Subcultures were done if necessary. Visible colonies were identified by standard microbiological methods (colonial morphology, gram stain, catalase test, oxidase test, clumping factor/coagulase test) and by matrix assisted laser desorption and ionization/time-of-flight (MALDI/TOF) mass spectrometry if necessary. In case of detection of *Staphylococcus aureus* strains, antibiotic susceptibility testing was performed by the disk-diffusion method according to the Clinical and Laboratory Standards Institute (CLSI). Evaluation also included counting of colony forming units (CFUs).

### Statistical Analysis

Statistical analysis was performed using dedicated software (SPSS Statistics v20, IBM, Armonk, USA). Microbial contamination (CFUs) of the surface of the device (ROI A, B and C; ROI A + ROI B + ROI C) was assessed each day before the device was handed over to the first patient and after the last patient of each day returned the device to the study assistant and was compared with the microbial contamination of the outside of the used plastic bags (ROI A, B and C; ROI A + ROI B + ROI C) of each day. Mann-Whitney U test was applied for the statistical workup because the data are not normally distributed and non-dependent. P-values < 0.05 were considered as significant, p-values < 0.001 were considered as highly significant. The statistical power was calculated retrospectively (π = 1.0).

## Results

### Patients

115 patients were included in this study. 15 participants had to be excluded as they had no waiting time and did not use the tablet. No patient recalled the agreement. Hence, 100 patients consisting of 50 females with a mean age of 51.6 ± 16.3 years and 50 males with a mean age of 46.7 ± 21.4 years successfully participated. 85% indicated a frequent use of a personal computer, while 44% and 15% indicated a frequent use of a smartphone or a tablet computer, respectively (Table 1). No patient reported medical problems that might have hindered them using the device (e.g. sensory problems in the fingertips). Patients used the device for 23 ± 17 minutes. Patients were recruited consecutively within a period of 11 days. We only excluded patients who were sedated nor mentally affected. 4 patients needed assistance while they were using the device. The study assistant provided help with a custom made instruction manual. In one case the study assistant needed to touch the device with sterile gloves.

**Table 1:** Experience of the participants with a personal computer (PC) and touchscreen operated devices (n  =  100).

	Do you use a tablet computer (iPad, etc.)?	Do you use a smartphone?	Do you use a PC?
**Never**	80	55	9
**Occasionally**	4	0	5
**Often**	6	10	40
**Very often**	9	34	45
**Not specified**	1	1	1

### Contamination of Unused Plastic Bags

None of the 4 randomly selected unused plastic bags did show any bacterial contamination (0 colony forming units, CFUs).

### Contamination of the Outside of Used Plastic Bags and of the Surface of the Tablet

**Figure 3:**
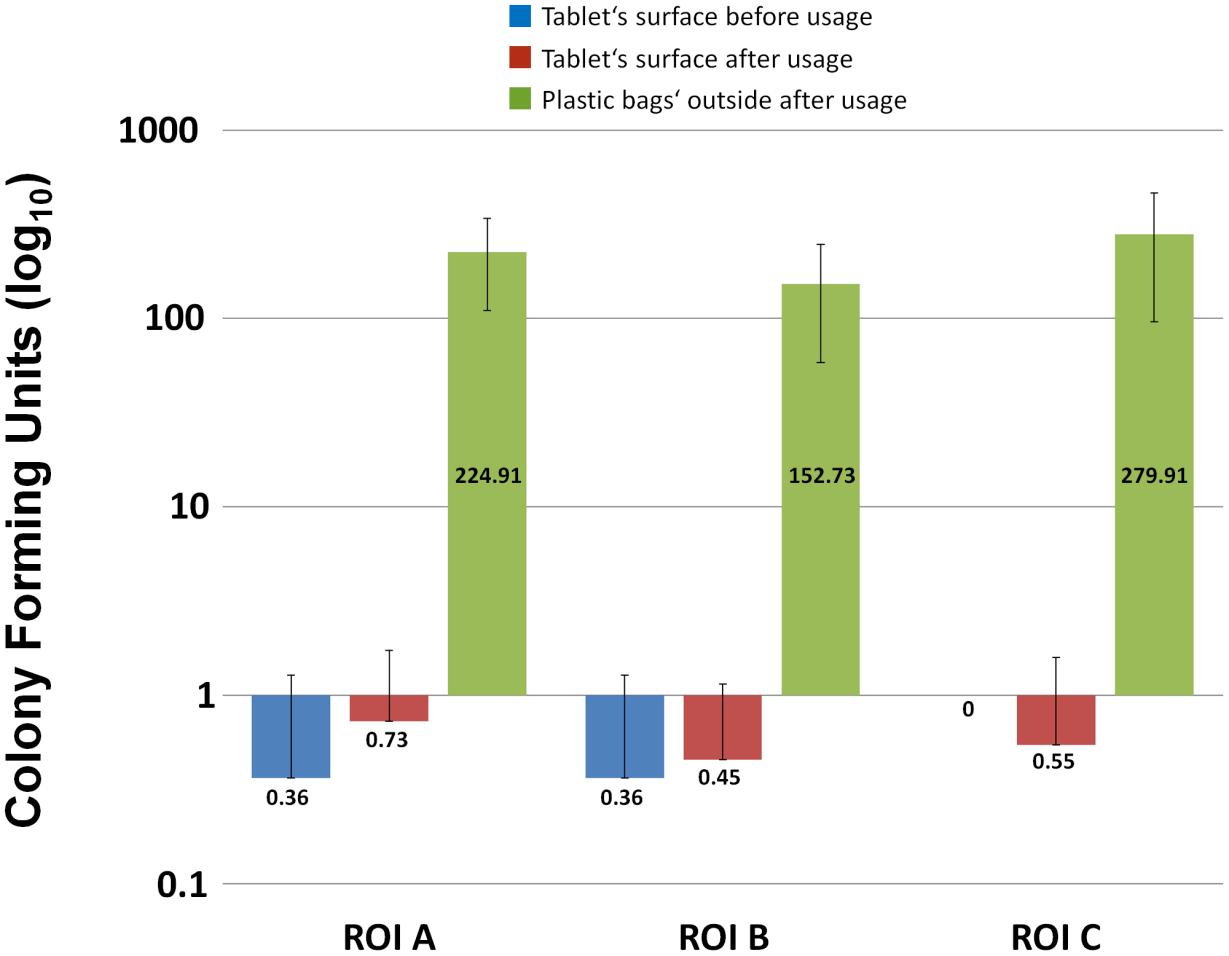
Microbial contamination of the surface of the tablet and of the outside of the used plastic bags. Logarithmic presentation of the mean microbial contamination (colony forming units, CFUs) of the surface of the tablet computer before the initial and after the final use of each day and of the outside of the plastic bags of each day. Mean values ± standard deviations are shown. Samples were taken from three defined regions of interest (ROIs; ROI A  =  central touchscreen, ROI B  =  home button and its adjacent area, ROI C  =  upper central backside).

The mean CFU amount on the outside of the used plastic bags of a day (ROI A + ROI B + ROI C) was 657.55 ± 368.46. Mean CFU amounts on ROI A, B and C were 224.91 ± 114.67, 152.73 ± 94.37 and 279.91 ± 183.66 (Table 2, Figure 3). The degree of contamination of the used plastic bags varied strikingly. In some samples 0 CFUs were detected (ROI A [3×], B [5×] and C [1×]), whereas other samples showed 150 or more CFUs per ROI (A and B [1× each], C [5×]). Considering the local distribution of the bacterial CFUs, ROI C was more frequently contaminated (42.6% of total CFUs) than ROI A (34.2% of total CFUs) and ROI B (23.2% of total CFUs).

**Table 2:** Microbial contamination (colony forming units, CFUs) of the outside of the plastic bags (ROI A/B/C/total) and of the surface of the tablet (ROI A/B/C/total) after a one-day usage.

	Microbial contamination [CFUs] of the outside of the used plastic bags (ROI A/B/C/total)	Microbial contamination [CFUs] of the surface of the tablet (ROI A/B/C/total)
Day 1	229/82/295/606	0
Day 2	321/147/191/659	2/0/2/4
Day 3	101/94/164/359	1/0/0/1
Day 4	312/175/401/888	0/1/0/1
Day 5	110/67/198/375	0
Day 6	191/113/370/674	0/1/0/1
Day 7	242/225/213/680	3/2/0/5
Day 8	478/379/751/1608	0
Day 9	182/211/263/656	1/1/1/3
Day 10	220/140/172/532	1/0/3/4
Day 11	88/47/61/196	0
Mean ± standard deviation	224.91 ± 114.67/152.73 ± 94.37/279.91 ± 183.66/657.55 ± 368.46	0.73 ± 1.01/0.45 ± 0.69/0.55 ± 1.04/1.73 ± 1.90

Microbial contamination of the surface of the tablet was significantly lower than the contamination of the outside of the used plastic bags (ROI A, B and C; ROI A + ROI B + ROI C; p < 0.001). ROI  =  Region of interest.

After the disinfection of the surface of the tablet and before wrapping it and handing it over to the first patient a mean contamination of 0.36 ± 0.92 CFUs was found on ROI A and B. ROI C was not contaminated (0 CFUs) (Table 3, Figure 3).

**Table 3:** Microbial contamination (colony forming units  =  CFUs) of the surface of the tablet computer was determined twice every day, before initial and after final usage (100 users in 11 days).

	Microbial contamination of the surface of the tablet before usage		Microbial contamination of the surface of the tablet after usage	
	Min/Max	Mean/SD	Min/Max	Mean/SD
ROI A [CFU]	0/3	0.36 ± 0.92	0/3	0.73 ± 1.01
ROI B [CFU]	0/3	0.36 ± 0.92	0/2	0.45 ± 0.69
ROI C [CFU]	0/0	0	0/3	0.55 ± 1.04

Samples were taken from three defined regions of interest (ROIs; ROI A  =  central touchscreen, ROI B  =  home button and its adjacent area, ROI C  =  upper central backside).

After the final patient of each day returned the device, mean contamination of the surface of the tablet was 1.73 ± 1.90 CFUs. 0.73 ± 1.01 CFUs on ROI A, 0.45 ± 0.69 CFUs on ROI B and 0.55 ± 1.04 CFUs on ROI C (Table 2 and 3, Figure 3). The number of CFUs on the surface of the tablet after one-day usage was significantly lower than the number of CFUs on the outside of the used plastic bags (ROI A, B and C; ROI A + ROI B + ROI C; p < 0.001). All bacterial species detected on the surface of the tablet belonged to the physiological mixed skin flora.

### Classification of Bacteria on the Outside of Used Plastic Bags

A total of 12 different types of bacteria were detected, which can be divided into the following groups (Table 4).

**Table 4:** Classification, prevalence and medical relevance of the bacteria found on the outside of used plastic bags. Prevalence [%] is shown in brackets.

	Classification of the bacteria found on the outside of used plastic bags					Medical relevance	
	Skin flora	Skin	Fecal	Oral	Environmental contaminants	Non-pathogenic ^(1)^	Potentially pathogenic
**Enterobacter cloacae [1]**			X				X
**Pantoea agglomerans [1]**			X				X
**Staphylococcus aureus [1]**		X					X
**Coagulase-negative staphylococci [100]**	X	X				X	
**Micrococci [66]**	X	X				X	
**Corynebacteria [68]**	X	X				X	
**Staphylococcus lugdunensis [1]**	X					X	
**Acinetobacter ursingii [1]**	X				X	X	
**Acinetobacter lwoffii [2]**	X				X	X	
**Aerobic spore-formers [64]**					X	X	
**Alpha-hemolytic Streptococci [2]**				X		X	
**Streptococcus sanguinis [1]**				X		X	

(1) unless inoculated into wounds or into the blood stream via catheters.

#### Non-Pathogenic Physiological Flora and Environmental Contaminants

The identification of the colonies mostly yielded gram-positive bacteria, such as coagulase-negative staphylococci, micrococci and *Corynebacterium* species. These bacteria are part of the normal flora of the skin and are usually classified as non-pathogenic, unless inoculated into wounds or into the blood stream via catheters. Furthermore *Streptococcus* species (e. g. *Streptococcus sanguinis*, unidentified alpha-hemolytic *Streptococcus* species), *Bacillus* species and *Acinetobacter* species could be identified. Whereas *Streptococcus sanguinis* and alpha-hemolytic streptococci can be considered as normal inhabitants of the human oral cavity, *Bacillus* species are common and wide-spread environmental contaminants. *Acinetobacter* (A.) are gram-negative bacteria that emerged as a significant nosocomial pathogens during the last decade; among the more than thirty species *A. baumannii/A. baumannii*-complex has the greatest clinical relevance. Infections by other *Acinetobacter* species, like *A. ursingii* or *A. lwoffii*, which were isolated in this study, are relatively unusual. Depending on the species *Acinetobacter* can be found on human skin, as oropharyngeal commensal or in the environment.

#### Potentially Pathogenic

This group consists of fecal bacteria and bacteria that are linked to nosocomial, i.e. hospital-acquired infections.

*Staphylococcus aureus*, *Enterobacter cloacae* and *Pantoea agglomerans* were identified. *Staphylococcus aureus* is known as a wide-spread nosocomial pathogen and as a community-associated pathogen; the strain isolated in this study showed susceptibility against oxacillin and therefore was not a methicillin-resistant *Staphylococcus aureus* (MRSA). *Enterobacter cloacae* and *Pantoea agglomerans* are gram-negative rods belonging to the Enterobacteriaceae. Their natural habitat is either the gastrointestinal tract of men or animals or the environment (soil, plants). Nosocomial infections with these pathogens have been reported.

Yeasts and moulds were not detected in this study.

### User Satisfaction and Functionality of the Touchscreen of a Plastic Bag Covered Tablet

5 of 115 patients tried to unpack the tablet after the device was handed over, what however was noticed and stopped by the study assistant. In two cases the plastic bag showed severe scratches after usage. Two patients reported problems with the performance of the device (1× lost internet connection, 1× not specified), while 94 patients did not experience any technical problems and 4 patients did not answer this question. 12 patients felt uncomfortable handling the tablet through the plastic cover, while 85 patients did not find the plastic cover irritating and three patients failed to answer this question. 11 patients reported problems with the functionality of the touchscreen, whereas 86 patients did not mention such a problem and three did not answer this question. Interestingly, all patients complaining about a disturbing plastic bag or impaired touchscreen functionality did not have any experience with touchscreen operated devices (Table 5).

**Table 5:** Convenience for the operators and functionality of the touchscreen using a plastic bag-covered tablet computer dependent on the experience of participants with touchscreen operated devices (n  =  100).

	Patients who used a touchscreen operated device before	Patients who never used a touchscreen operated device before
**The plastic bag impairs the user satisfaction**	0	12
**The plastic bag impairs the functionality of the touchscreen**	0	11

81% of the patients rated the availability of a tablet computer during the waiting period as “very good”, 9% as “good”, 6% as fair and 3% as “poor”. 1% of the patients did not answer this question.

## Discussion

In this study we could show that when using touchscreen operated portable devices in a clinical setting the microbial contamination can be significantly reduced by wrapping the device with customized single-use plastic bags. We detected a minor contamination of the surface of the tablet computer after frequent usage of the wrapped device. In most cases no CFUs were detected and the maximum of contamination after a one-day usage was 5 CFUs of physiological skin flora bacteria. The custom-made plastic bags seem to be practicable despite not being a sterile product as they did not show any contamination with bacteria prior to their use.

The microorganisms isolated on the outside of the used plastic bags were mostly members of the physiological flora of the human skin or of the oral cavity or non-pathogenic environmental bacteria. However in some cases potentially pathogenic bacteria, such as *Staphylococcus aureus*, or Enterobactericeae (e.g. *Enterobacter cloacae* or *Pantoea agglomerans*) were detected. These findings indicate that the use of tablet computers in a clinical setting, even for a short period of time, poses the risk of deposition and transmission of pathogenic microorganisms.

A recently published study predicts that a majority of American physicians most likely will use mobile devices and especially tablet computers in the near future [7]. Many studies show various applications of tablet computers in a clinical setting, such as carrying out the documented patient briefing with an iPad app [14], supporting doctors during patient consultations [15] or during hospital wards [16].

The presented study aimed to address hygienic concerns associated with the use of touchscreen operated devices in a clinical setting and to outline a procedure that is compatible with current hygiene standards. To carefully consider hygienic requirements is of particular importance, because infectious diseases and nosocomial pathogens increasingly cause problems in hospital settings. One aspect is the multi-drug resistance of certain bacteria. In addition, many relevant pathogens can persist on dry inanimate surfaces for weeks or even months. In hospitals, frequently touched surfaces are often contaminated with a variety of relevant pathogens [1], [2], [17]. Especially mobile devices that are touchscreen operated and usually handed over from person to person perfectly fulfill the conditions to serve as vehicles for transmission.

The results show that even a short contact with the device can lead to contamination and consequently to pathogen transfer. Kampf et al. reported that the compliance rate of healthcare workers concerning hand hygiene is around 50% [3]. Usually, patients are not encouraged to perform hand disinfection at all. However, hands are known to be one of the major vectors for pathogen transmission [4]–[6]. The combination of a low compliance concerning hand hygiene and the rising number of mobile and immobile devices that are often operated with a touchscreen or a touch pad leads to a substantial risk for pathogen colonization and subsequent infectious diseases.

The manufacturer Apple does not provide a recommendation how to disinfect the device. Apple recommends: (1) use only a soft, slightly damp, lint-free cloth; and do not use abrasive cloths, towels, paper towels, and similar items that may cause screen damage; (2) disconnect the product from any external power source, unplug all cables, and turn off the device; and (3) avoid getting moisture in openings; do not use window cleaners, household cleaners, aerosol sprays, solvents, alcohol, ammonia, or abrasives; and do not spray cleaners directly onto the device [12]. This recommendations show that the device was not developed for the hospital health care setting and that disinfection is very limited. Hence, the proposed protection could offer an alternative to prepare tablet computers for their usage in a clinical setting.

There are few data available on how to effectively clean/disinfect touchscreen operated devices without causing damage and there can be found some recommendations to place waterproof or water resistant barriers over the device [18]–[20]. However, to our knowledge, no data are available on a microbiological workup of the proposed approach (wrapping the device with a single-use plastic bag).

It is worth to mention that there is a possibility to establish a humid space inside a plastic bag. Such a milieu between the tablet and the plastic bag can be a good nursing ground for pathogens. However, we assume that it is not likely to create a humid space inside the plastic bag when bagging a dry tablet and when changing the bag regularly (in this study after each patient; average use: 23 ± 17 minutes). Our results show, that under these circumstances there is minor contamination or bacterial growth on the surface of the tablet during a one-day usage (Table 3, Figure 3).

Regarding the tolerance of the patients with the plastic bags, some issues need to be addressed. Roughly 4% of the patients tried to unwrap the tablet or scratched/damaged the plastic bag. Therefore, a short notice not to unwrap the sealed device or to damage the cover seems necessary. Furthermore, 12% of the patients felt uncomfortable using the tablet with the surrounding plastic bag and 11% of the patients complained about an impaired functionality of the touchscreen. However, all these patients indicated that they have never used a tablet computer or a smartphone before. An explanation for that kind of criticism could be that these patients are just not familiar with touchscreen operated devices. It is well known that even though touchscreens can be used quite intuitively, there is some kind of a learning curve. Interestingly, none of the touchscreen-experienced patients complained about an impaired user satisfaction or touchscreen functionality. 2% of the patients reported problems with the performance of the device, such as system crashes which are obviously not related with the wrapping of the tablet computer. These numbers imply that the covering bags hardly impaired the user satisfaction and the functionality of the touchscreen.

Our study faces some limitations that suggest directions for future work.

The study population is not representative for the clientele of the hospital because only patients who were referred to the Department of Radiology for an MRI examination were included. In order to ensure proper handling of the device, included patients were not mentally or physically affected.

Further, we did not specifically investigate the durability of the selected plastic bags. The bag was replaced after each patient and although there was apparent scratching of the surface of the bag in some cases we did not notice significant contamination of the subjacent surface of the tablet. Nevertheless, it would be interesting to further consider the durability of the plastic bag under varying conditions.

## Conclusions

Portable tablet computers get severely contaminated during usage in a clinical setting Microbial contamination of these devices can be significantly reduced by wrapping the device with a customized single-use plastic bag. This is a promising approach to prepare tablets for their usage in a clinical setting because according to the recommendations of the manufacturer, disinfection options are very limited. Wrapping did not significantly impair the user satisfaction or the functionality of the touchscreen when patients were somewhat experienced with touchscreens.

## Supporting Information

Summary Points S1 What was already known and what this study has added.
(DOCX)
