# Supplementary material for: Practicability of Hygienic Wrapping of Touchscreen Operated Mobile Devices in a Clinical Setting
Source: PLoS One. 2014 Sep 2;9(9):e106445. doi: 10.1371/journal.pone.0106445 (PMC4152284; doi:10.1371/journal.pone.0106445)
Supplement: Summary Points S1 — What was already known and what this study has added. (DOCX) [file pone.0106445.s001.docx]

**Summary Points S1**

**What was already known**

- Tablet computers are becoming more and more popular for a variety of clinical or clinical related tasks.
- Tablet computers will likely be used by a majority of American physicians in the near future.
- Touchscreen operated mobile devices potentially facilitate the distribution of pathogens.
- Disinfection of these devices with a leaky, non-protected case does not seem to be straightforward.

**What this study has added**

- Tablet computers get severely contaminated with various, partially potentially pathogenic bacteria during usage in a clinical setting.
- Microbial contamination of the tablet computer can be significantly reduced by wrapping the device with a customized single-use plastic bag.
- Wrapping hardly impairs operability or user satisfaction of touchscreen operated devices. 11 % of the patients reported problems with the functionality of the touchscreen. These patients indicated that they had never used a touchscreen operated device (tablet computer, smartphone, etc.) before.
- 81 % of the patients rated the availability of a tablet computer during the waiting period as “very good”.
